# Inherited variations in human pigmentation-related genes modulate cutaneous melanoma risk and clinicopathological features in Brazilian population

**DOI:** 10.1038/s41598-020-68945-9

**Published:** 2020-07-22

**Authors:** Gustavo Jacob Lourenço, Cristiane Oliveira, Benilton Sá Carvalho, Caroline Torricelli, Janet Keller Silva, Gabriela Vilas Bôas Gomez, José Augusto Rinck-Junior, Wesley Lima Oliveira, Vinicius Lima Vazquez, Sergio Vicente Serrano, Aparecida Machado Moraes, Carmen Silvia Passos Lima

**Affiliations:** 1 Laboratory of Cancer Genetics, Faculty of Medical Sciences, University of Campinas, Campinas, São Paulo Brazil; 2 Department of Statistics, Institute of Mathematics, Statistic, and Computer Science, University of Campinas, Campinas, São Paulo Brazil; 3 Clinical Oncology Service, Department of Internal Medicine, Faculty of Medical Sciences, University of Campinas, Rua Alexander Fleming, 181, Cidade Universitária “Zeferino Vaz”, Barão Geraldo, Campinas, São Paulo Brazil; 4 A.C. Camargo Cancer Center, São Paulo, São Paulo Brazil; 5 Melanoma and Sarcoma Surgery Department, Barretos Cancer Hospital, Barretos, São Paulo Brazil; 6 Department of Medical Oncology, Barretos Cancer Hospital, Barretos, São Paulo Brazil

**Keywords:** Melanoma, Cancer genetics

## Abstract

Ultraviolet light exposure and cutaneous pigmentation are important host risk factors for cutaneous melanoma (CM), and it is well known that inherited ability to produce melanin varies in humans. The study aimed to identify single-nucleotide variants (SNVs) on pigmentation-related genes with importance in risk and clinicopathological aspects of CM. The study was conducted in two stages. In stage 1, 103 CM patients and 103 controls were analyzed using Genome-Wide Human SNV Arrays in order to identify SNVs in pigmentation-related genes, and the most important SNVs were selected for data validation in stage 2 by real-time polymerase-chain reaction in 247 CM patients and 280 controls. *ADCY3* c.675+9196T>G, *CREB1* c.303+373G>A, and *MITF* c.938-325G>A were selected for data validation among 74 SNVs. Individuals with *CREB1* GA or AA genotype and allele “A” were under 1.79 and 1.47-fold increased risks of CM than others, respectively. Excesses of *CREB1* AA and *MITF* AA genotype were seen in patients with tumors at Clark levels III to V (27.8% versus 13.7%) and at III or IV stages (46.1% versus 24.9%) compared to others, respectively. When compared to others, patients with *ADCY3* TT had 1.89 more chances of presenting CM progression, and those with *MITF* GA or AA had 2.20 more chances of evolving to death by CM. Our data provide, for the first time, preliminary evidence that inherited abnormalities in *ADCY3*, *CREB1*, and *MITF* pigmentation-related genes, not only can increase the risk to CM, but also influence CM patients’ clinicopathological features.

## Introduction

Cutaneous melanoma (CM) is the most deadly form of skin cancers^1^. Ultraviolet (UV) light exposure and individual pigmentation features are well-established host risk factors for CM^1^, and tumor depth and stage are the most important hallmarks of CM prognosis^2^. Moreover, previous studies have shown that inherited genetic variants modulate CM risk^3^ and outcome^4^.

**Figure 1:**
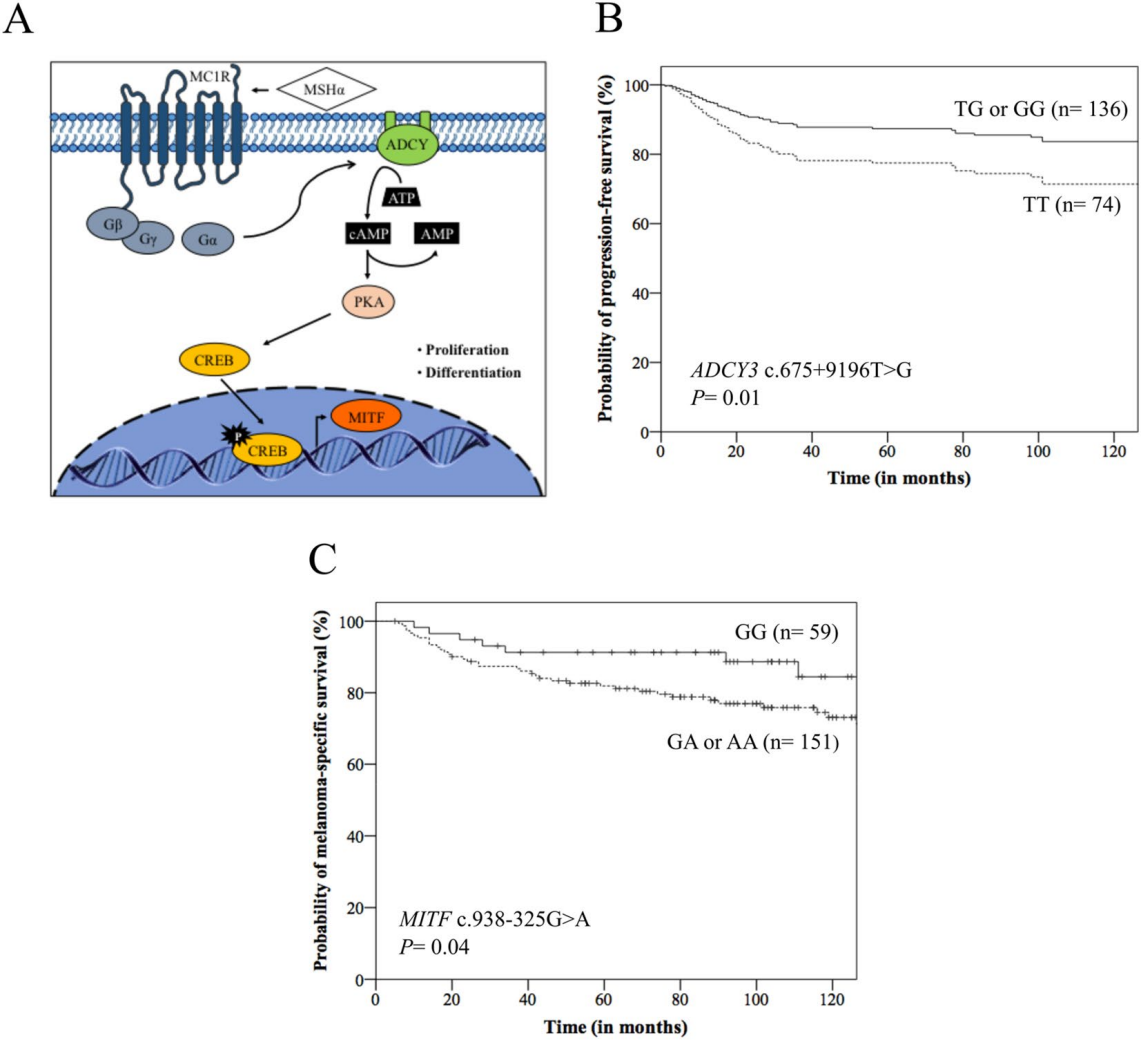
(**A**) Pigmentation regulation by alpha-melanocyte stimulating hormone (MSHα) and G-proteins from melanocortin receptor 1 (MC1R): MSHα/MCR1 can trigger the activation of the adenylate cyclase (ADCY) and 3′–5′-cyclic adenosine monophosphate AMP (cAMP). The cAMP signals activate the protein kinase A (PKA) that phosphorylates and activates cAMP responsive element binding protein (CREB) transcription factor, which induces the expression of melanocyte inducing transcription factor (MITF) and induction of proliferation and differentiation of melanocytes. G-proteins: β, γ, and α; ATP: adenosine triphosphate. (**B**) Kaplan–Meier (K-M) curves for progression-free survival according to *ADCY3* c.675+9196T>G genotypes, where patients with TT genotype presented lower survival than those with TG or GG genotype. (**C**) K-M curves for melanoma-specific survival according to *MITF* c.938-325G>A genotypes, where patients with GA or AA genotype presented lower survival than those with GG genotype.

Melanocytes absorb UV radiation and survive to considerable genotoxic stress, and the main genetic mechanism involved in CM development alters the control of skin pigmentation^1,5,6^. Sunlight exposure induces the post-inflammatory hyperpigmentation system by the melanocortin-1 receptor (MC1R), and melanocytes express MC1R that regulates the quality and quantity of their melanin production^7^. The α-melanocyte stimulating hormone (MSHα) activates the membrane associated enzyme adenylate cyclase (ADCY), increasing 3′-5′-cyclic adenosine monophosphate (cAMP) levels; the increased cAMP signals activate protein kinase A (PKA) that activates cAMP responsive element binding protein (CREB)^5,6,8^. CREB is a transcription factor that regulates the expression of melanocyte inducing transcription factor (MITF) and consequently, proliferation and differentiation of melanocytes and melanin synthesis^5,6,8^ (Fig. 1A).

On the other hand, melanogenesis generates mutagenic intermediates (quinones and semiquinones), neutralizes reactive oxygen species, eliminates free radicals, and modifies cell metabolism through the stimulation of aerobic glycolysis generating a hypoxic environment^9,10^, making melanoma cells resistant to chemo-, radio-, photo- and immunotherapy^9,11^. Brożyna et al. showed that nonpigmented cells were significantly more sensitive to gamma rays than pigmented cells^12^. Melanogenesis induction is also related to significant up-regulation of hypoxia-inducible factors (HIF) and these factors are key master regulators of cellular metabolism and therapeutic resistance^11,13^, contributing to the increased aggressiveness of melanoma and shorter survival time of patients with pigmented metastatic melanoma than the ones with amelanotic lesion^11^.

Genome-wide association studies (GWAS), conducted particularly in Caucasians, have identified single nucleotide variants (SNVs) associated with CM risk, many of which in human pigmentation genes, such as *MC1R*, solute carrier family 45 member 2 (*SLC45A2*) and tyrosinase (*TYR*)^14–17^. The most GWAS have identified SNVs located in non-coding regions of the genome which can affect gene regulatory sequences, and consequently the gene expression^18^. SNVs located in introns can also alter the precursor RNA messenger (pre-mRNA) splicing process^19^ or the binding sites for regulatory proteins splicing^20^, influencing the efficiency of splicing or inducing alternative splicing, and an intronic variant of poly(ADP-ribose) polymerase number 1 (*PARP1*) was associated with increased CM risk in Caucasians^21^.

The Brazilian population is highly heterogeneous, consisting of indigenous Amerindians and immigrants from Europe, Africa, and Asia^22^. Since other SNVs in genes with equal or even greater importance in melanogenesis may not have been selected in the previous analyzed populations, we conducted an association study in patients with CM and healthy controls from Brazil, and identified three SNVs of MC1R pathway, *ADCY3* c.675+9196T>G, *CREB1* c.303+373G>A and *MITF* c.938-325G>A in association with tumor risk and clinicopathological features.

## Material and methods

### Study population

This association study was conducted in two stages. In stage 1, 103 CM patients and 103 controls were analyzed with the purpose of identifying SNVs on pigmentation-related genes with importance in CM risk, and in stage 2 the most important SNVs were selected for data validation in 247 CM patients and 280 controls.

All CM patients were diagnosed at the Clinical Oncology and Dermatology Services of University of Campinas, A.C. Camargo Cancer Center, and Barretos Cancer Hospital between April 2000 and May 2018. Patients diagnosed with the unknown primary site tumors and those with tumors located in mucous were excluded from the study. The control group was compound by blood donors seen at the Hematology and Hemotherapy Center of University of Campinas in the same period. The study was approved by the local Ethical Committees of both Institutions (numbers: 424/20016 and 1.438.601). All procedures were carried out according to the Helsinki Declaration, and appropriate informed consent was obtained.

### Data and specimen collection

Clinical information of individuals (age at diagnosis, gender, skin color, skin phototype, sun exposure, type of sun exposure, and number of nevi) was obtained by specific questionnaires. Skin phototype was defined using reported criteria^23^. Individuals exposed to the sun for more than 2 h per day and for more ten years were considered positive for sun exposure^23^. Sun exposure was classified as intermittent in cases of recreational activities performed less than 50% of the week or holidays, or chronic, activities at home or work under sunlight exposure during more than 50% of the time^24^.

The diagnosis of CM was established by histopathological evaluation of tumor fragments embedded in paraffin and stained with hematoxylin and eosin. Pathological aspects of the tumor (tumor location, histological type, Breslow thickness, Clark level, and tumor stage) were obtained from medical records of patients^25^. Tumor stage was identified using the TNM classification of the American Joint Committee on Cancer, where T describes the size of tumor, N describes spread of tumor to nearby lymph nodes, and M describes distant metastasis^2^. Patients with desmoplastic, acro-lentiginous and amelanotic melanomas were excluded from the study.

Surgical excision (n = 217) was the primary treatment for patients with localized tumor^26^. Sentinel lymph node biopsy (n = 41) was recommended in patients with tumor measuring more than 1 mm (mm) and lymphadenectomy (n = 24) was performed in patients with clinically positive lymph nodes or lymph nodes with tumor infiltration on histopathological evaluation. Patients with operable single metastasis or relapse (n = 30) underwent surgical resection^27^. Those patients with inoperable relapse or multiple metastases (n = 30) received chemotherapy with dacarbazine^28^. Radiotherapy was also used in the local treatment of patients with surgical impossibility (n = 4), particularly in bleeding lesions, bone or brain metastases^29^.

### Stage 1: screening of SNVs, candidate genes choice and SNVs selection

DNA from leukocytes of peripheral blood of CM patients and controls were genotyped for a total of 906,660 SNVs using the Affymetrix Genome-Wide Human SNV Arrays 6.0 (AFFYMETRIX, USA), according to the manufacturer’s recommended protocols. The intensities resulting from the arrays scanning process were made available via CEL files, one per DNA sample with total quality control higher than 90% (AFFYMETRIX, USA). Tools from the Bioconductor (https://www.bioconductor.org) were used to process the CEL files. The genotyping was performed applying the corrected robust linear mixture model (crlmm) algorithm^30^.

The genes previously reported as involved in the pigmentation pathway were selected for study. The pathway analysis was performed using the Database for Annotation, Visualization and Integrated Discovery (https://david.ncifcrf.gov^31^ and Kyoto Encyclopedia of Genes and Genomes pathway maps (https://www.kegg.jp)^32^.

Each pigmentation related-gene was analyzed using the in silico method by the Human Splicing Finder algorithm (version 3.1) (https://www.umd.be/HSF3/index.html^33^ in order of identifying SNVs in splicing regulatory sequences. For analysis, wild-type (ancestral) allele was taken as reference. SNVs showing deviation from the HWE and those with the minor allele frequency less than 10% were excluded from the selection^34^. SNVs that potentially alter expression or function of the encoding proteins^13,14^ were selected for further validation.

### Stage 2: validation of selected SNVs in risk and characteristics of melanoma

DNA from leukocytes of peripheral blood of CM patients and controls was analyzed by real-time polymerase chain reaction with TaqMan SNV genotyping assays (APPLIED BIOSYSTEMS, USA) for *ADCY3* (rs11900505, assay ID: C_7868411_20), *CREB1* (rs10932201, assay ID: C_2859093_20) and *MITF* (rs7623610, assay ID: C_29012190_10) SNVs, following manufacturer instructions. Twenty percent of genotype determinations were carried out twice in independent experiments with 100% of concordance.

Frequencies of *ADCY3* c.675+9196T>G, *CREB1* c.303+373G>A and *MITF* c.938-325G>A genotypes, isolated and in combination, were analyzed in patients and controls, and in patients stratified by clinicopathological features.

### Gene expression by quantitative PCR

Total RNA was obtained from leukocytes of peripheral blood of CM patients and controls with distinct genotypes of *ADCY3* (16 and 18 with TT genotype, 16 and 19 with TG, and seven and 19 with GG, respectively), *CREB1* (14 and 10 with GG genotype, 16 and 25 with GA, 9 and 18 with AA, respectively) and *MITF* (15 and 28 with GG genotype, 16 and 23 with GA, 6 and 23 with AA, respectively) with TRIzol reagent (LIFE TECHNOLOGIES, USA).

cDNA was generated using Maxima First Strand cDNA Synthesis kit reagents (LIFE TECHNOLOGIES, USA) following manufacturer’s instructions. The experiments were performed using SYBR Green PCR Master Mix reagents (APPLIED BIOSYSTEMS, USA) and specific primers for *ADCY3* (forward: 5′-TCATCTCCGTGGTCTCCTG-3′ and reverse: 5′-CACAGGTAGAGGAA-GACGTTG-3′), *CREB1* (forward 5′-CTAGTACAGCTGCCCA-ATGG-3′ and reverse: 5′-AGTTG-AAATCTGTGTTCCGG-3′), and *MITF* (forward: 5′-AGTCTGAAGCAAGAGCACTG-3′ and reverse: 5′-GCGCATGTCTGGATCATTTG-3′) genes, in triplicate per sample, and a control without template were included in each plate. The relative expression level of genes was normalized to that of reference housekeeping gene actin beta (forward: 5′-AGGCCAACCGCGAGAAG-3′ and reverse: 5′-ACAGCCTGGATAGCAACGTACA-3′) using 2^−DDCt^ cycle threshold method^35^. Twenty percent of samples had evaluation repeated in separate experiments with 100% agreement. The results were expressed in arbitrary units (AUs).

### Statistical analysis

Association between disease statuses, CM patients versus controls, and genotypes for study’s stage 1 was performed using logistic regression model, and analyses were adjusted by age at diagnosis, skin color, and sun exposure. SNVs that presented raw *p*-values below the 0.001 thresholds were selected for further inspection. These analyses were implemented in R software (version 3.3.0) (https://www.r-project.org).

The Hardy–Weinberg equilibrium (HWE) was tested using chi-square (χ^2^) statistics for the goodness-to-fit test, and logistic regression model served to obtain age, skin color, sun exposure, and number of nevi status-adjusted crude odds ratios (ORs) with 95% confidence intervals (CI) in comparisons evolving patients and controls for study’s stage 2. To evaluate the robustness of risk estimates, the false discovery rate (FDR) was computed, which reflects the expected ratio of false-positive findings to the total number of significant findings; the differences revealed were considered statistically significant at FDR values < 0.05^25^. χ^2^ and Fisher’s exact tests were used to evaluate associations between clinicopathological features and genotypes of selected SNVs. Bonferroni method was used to adjust values of multiple comparisons in patients stratified by tumor aspects^36^. For *ADCY3*, *CREB1* and *MITF* expression analysis, data sets were probed for normality using Shapiro–Wilk’s test. Because data sets assume normal distribution, analysis of variance performed comparisons of groups^36^.

For survival analysis, the progression-free survival (PFS) was calculated from the date of surgery until the date of first recurrence, or the date of progression of disease, or the date of death by any cause, or the date of last follow-up. The melanoma-specific survival (MSS) was calculated from the date of diagnosis until the date of death by the disease or last follow-up. PFS and MSS were calculated using Kaplan–Meier estimates, and differences between survival curves were analyzed by log-rank test^25^. The impact of age at diagnosis, gender, tumor location, Breslow thickness^37^, Clark level, TNM stage and genotypes of each analyzed SNV in survival of patients were evaluated using univariate Cox proportional hazards ratio (HR) regression. At a second time, all variables with *p* < 0.20 were included in the multivariate Cox regression. The significant results of Cox analysis were internally validated using a bootstrap resampling study to investigate the stability of risk estimates (1,000 replications)^25^.

All tests were done using the SPSS 21.0 software (SPSS INCORPORATION, USA). Significance was two sided and achieved when *p* values were ≤ 0.05.

## Results

### Study population

The clinicopathological features of patients and the clinical features of controls included in stage 1 and stage 2 of the study are presented in Table 1. Controls were younger than patients, and CM patients presented more white skin color, referred more sun exposure and presented more nevi than controls, and all differences were corrected in comparisons involving patients and controls by appropriate statistical analysis. Similar clinicopathological features were observed in patients and controls analyzed in both stages of the study.

**Table 1:** Clinicopathological aspects of patients with cutaneous melanoma and clinical features of controls used in screening (stage 1) and validation (stage 2) of single nucleotide variants in human pigmentation-related genes. *The numbers of individuals were not the same included in the study because no consistent information could be obtained from some individuals. NC: values were not computed. *P* values describe differences between patients and controls and values < 0.05 are presented in bold letters.

Characteristics	Stage 1			Stage 2		
	Patients n (%)	Controls n (%)	*p* value	Patients n (%)	Controls n (%)	*p* value
**Age (years)**						
≤ 55	55 (53.4)	93 (90.3)	**< 0.0001**	125 (50.6)	229 (81.8)	**< 0.0001**
> 55	48 (46.6)	10 (9.7)		122 (49.4)	51 (18.2)	
**Gender**						
Male	61 (59.2)	55 (53.4)	0.48	130 (52.6)	145 (51.8)	0.84
Female	42 (40.8)	48 (46.6)		117 (47.4)	135 (48.2)	
**Skin color**						
White	97 (94.2)	80 (77.7)	**0.001**	231 (93.5)	231 (82.5)	**< 0.0001**
Non-white	6 (5.8)	23 (22.3)		16 (6.5)	49 (17.5)	
Phototype*						
I or II	58 (68.2)	53 (53.0)	0.05	154 (66.7)	164 (60.1)	0.12
III to VI	27 (31.8)	47 (47.0)		77 (33.3)	109 (39.9)	
Sun exposure*						
Yes	70 (82.4)	52 (52.0)	**< 0.0001**	196 (83.1)	126 (45.0)	**< 0.0001**
No	15 (17.6)	48 (48.0)		40 (16.9)	154 (55.0)	
Type of sun exposure*						
None or intermittent	32 (34.4)	76 (73.8)	**< 0.0001**	96 (44.2)	210 (75.0)	**< 0.0001**
Chronic	61 (65.6)	27 (26.2)		121 (55.8)	70 (25.0)	
Number of nevi*						
≤ 50	68 (80.0)	91 (97.8)	**0.0001**	183 (75.9)	260 (98.1)	** < 0.0001**
> 50	17 (20.0)	2 (2.2)		58 (24.1)	5 (1.9)	
**Tumor location**						
Head	16 (18.4)	NA	NC	46 (18.6)	NC	NC
Trunk	41 (47.1)	NA	NC	118 (47.8)	NC	NC
Upper limb	13 (14.9)	NA	NC	40 (16.2)	NC	NC
Lower limb	17 (19.6)	NA	NC	43 (17.4)	NC	NC
Histological type*						
Superficial spreading	37 (50.7)	NA	NC	119 (57.2)	NC	NC
Lentigo malign	9 (12.3)	NA	NC	26 (12.5)	NC	NC
Nodular	27 (37.0)	NA	NC	63 (30.3)	NC	NC
Breslow thickness (mm)*						
≤ 1.5	44 (46.8)	NA	NC	123 (52.8)	NC	NC
> 1.5	50 (53.2)	NA	NC	110 (47.2)	NC	NC
Clark level*						
I	14 (16.5)	NA	NC	31 (13.2)	NC	NC
II	12 (14.1)	NA	NC	42 (17.9)	NC	NC
III	27 (31.8)	NA	NC	60 (25.5)	NC	NC
IV	30 (35.3)	NA	NC	94 (40.0)	NC	NC
V	2 (2.3)	NA	NC	8 (3.4)	NC	NC
TNM stage*						
0	12 (13.8)	NA	NC	30 (12.3)	NC	NC
I	23 (26.4)	NA	NC	92 (37.7)	NC	NC
II	27 (31.0)	NA	NC	83 (34.0)	NC	NC
III	22 (25.3)	NA	NC	27 (11.1)	NC	NC
IV	3 (3.5)	NA	NC	12 (4.9)	NC	NC

### Screening of SNVs, candidate genes choice and SNVs selection

We found 12,495 new SNVs associated with CM risk; 6,497 (52.0%) of them were in introns, 5,928 (47.4%) in gene regulatory regions, and 70 (0.6%) in coding regions. The genome association data were deposited at Gene Expression Omnibus (GEO) database (https://www.ncbi.nlm.nih.gov/geo) with accession number GSE129890.

The most significant melanoma associated SNVs identified in stage 1 (*p* < 0.0001) are presented in Table S1 Supplement. Seventy-four SNVs in 28 human pigmentation-related genes were found to be involved with CM risk (Table S2 Supplement).

In accord with results of the in silico analysis, the variant allele “G” of *ADCY3* c.675+9196T>G may abolish a potential branch point site and an exonic splicing enhancer (ESE), and this variation may create a site of ligation for SRp55 and 9G8 splicing proteins. Besides, new sites for an exon-identity element (EIE) and an intron-identity element (IIE) may be created. The variant allele “A” of *CREB1* c.303+373G>A may abolish a splice donor site (5′ end of the intron), an exonic splicing silencer (ESS), an IIE site, and a binding site for the hnRNP A1, and this variation may create a new branch point, an EIE site, and a putative exonic splicing enhancer. The variant allele “A” of *MITF* c.938-325G>A may create a splice donor site, an ESS, and a binding site for the hnRNP A1, and this variation may break a potential branch point site, an EIE, and silencer motifs and an IIE site (Table S3 Supplement). *ADCY3* c.675+9196T>G, *CREB1* c.303+373G>A, and *MITF* c.938-325G>A SNVs were selected for analysis in stage 2 of the study due to their potential effects on encoding proteins^13,14^.

### Selected SNVs in risk and clinicopathological features of melanoma

Patient and control samples included in the stage 2 were in HWE at *ADCY3* c.675+9196T>G (χ^2^ = 0.58, *p* = 0.44; χ^2^ = 0.70, *p* = 0.40), and *CREB1* c.303+373G>A (χ^2^ = 0.08, *p* = 0.77; χ^2^ = 1.55, *P* = 0.21) loci, respectively. Controls’ samples (χ^2^ = 0.92, *p* = 0.33) but not patients’ samples (χ^2^ = 4.40, *p* = 0.03) confirmed the HWE at *MITF* rs7623610 locus.

*CREB1* GA or AA genotype and allele “A” were more common in patients than in controls; carriers of the above genotypes and allele were under 1.79 and 1.47-fold increased risks for CM than those with the GG genotype and allele “G”, respectively (Table 2). No associations between *ADCY3*, *CREB1* and *MITF* SNVs combined genotypes were seen in CM patients and controls (Table S4 Supplement).

**Table 2:** *ADCY3* c.675+9196T>G, *CREB1* c.303+373G>A, and *MITF* c.938-325G>A genotypes and alleles in 247 patients with cutaneous melanoma and 280 controls. OR: odds ratio adjusted by age, skin color, sun exposure, and number of nevi by multiple regression analysis; *CI* confidence interval; *P*^c^ values are *p* values corrected for multiple testing by the false discovery rate test. *P* and *p*^c^ values < 0.05 are presented in bold letters.

Genotype or allele	Patients N (%)	Controls N (%)	*P* value (*p*^c^ value)	OR (95% CI)
***ADCY3 c.675+9196T>G***				
TT	84 (34.0)	86 (30.7)	0.84 (0.84)	1.04 (0.66–1.64)
TG or GG	163 (66.0)	194 (69.3)		Reference
TT or TG	209 (84.6)	218 (77.9)	0.07 (0.12)	1.67 (0.95–2.93)
GG	38 (15.4)	62 (22.1)		Reference
Allele T	294 (59.5)	304 (54.3)	0.35 (0.35)	1.15 (0.85–1.56)
Allele G	200 (40.5)	256 (45.7)		Reference
***CREB1 c.303+373G>A***				
GG	68 (27.5)	110 (39.3)	**0.01** (**0.04**)	Reference
GA or AA	179 (72.5)	170 (60.7)		1.79 (1.14–2.82)
GG or GA	189 (76.5)	233 (83.2)	0.19 (0.28)	Reference
AA	58 (23.5)	47 (16.8)		1.43 (0.83–2.46)
Allele G	257 (52.0)	344 (61.4)	**0.01** (**0.04**)	Reference
Allele A	237 (48.0)	216 (38.6)		1.47 (1.08–2.00)
***MITF c.938-325G>A***				
GG	71 (28.7)	92 (32.9)	0.49 (0.55)	Reference
GA or AA	176 (71.3)	188 (67.1)		1.17 (0.74–1.85)
GG or GA	178 (72.1)	222 (79.3)	**0.02** (0.06)	Reference
AA	69 (27.9)	58 (20.7)		1.76 (1.07–2.89)
Allele G	249 (52.0)	344 (61.4)	0.06 (0.12)	Reference
Allele A	237 (48.0)	216 (38.6)		1.28 (0.84–1.94)

No associations of studied SNVs genotypes were seen in CM patients stratified by age, gender, and skin color (Table S5), phototype, sun exposure, type of sun exposure, and number of nevi (Table S6). However, *CREB1* AA genotype was more common in patients with tumors located in limbs than in head or trunk (31.7% versus 15.9%, *p* = 0.009) and tumors with Clark levels III to V than in those with tumors of I or II Clark levels (27.8% *versus* 13.7%, *p* = 0.012), and *MITF* AA genotype was more common in patients with III or IV tumor stage than in those with tumors at 0 to II stages (46.1% versus 24.9%, *p* = 0.007). These results were significant even after Bonferroni correction (corrected *p* value: 0.0125) (Table 3).

**Table 3:** *ADCY3* c.675 + 9196 T > G, *CREB1* c.303 + 373G > A, and *MITF* c.938-325A > G genotypes in 247 patients with cutaneous melanoma stratified by tumor features. Values are expressed as number and percentage. ^*^The numbers of patients were not the same included in the study (n = 247) because no consistent information could be obtained from some individuals. *P* values < 0.05 are presented in bold letters. ^**^Significant even after Bonferroni correction for multiple comparisons (corrected *p* value = 0.0125).

Genotypes	Histological type*		Breslow thickness (mm)*		Clark level*		TNM stage*	
	Head/trunk	Upper/lower limb	≤ 1.5	> 1.5	I or II	III to V	0 to II	III or IV
***ADCY3***								
TT	53 (32.3)	31 (37.4)	42 (34.1)	37 (33.6)	23 (31.5)	57 (35.2)	71 (34.6)	12 (30.8)
TG or GG	111 (67.7)	52 (62.6)	81 (65.9)	73 (66.4)	50 (68.5)	105 (64.8)	134 (65.4)	27 (69.2)
*P* value	0.18		0.93		0.58		0.64	
TT or TG	121 (83.4)	55 (87.3)	104 (84.5)	95 (86.4)	65 (89.0)	134 (82.7)	173 (84.4)	34 (87.2)
GG	24 (16.6)	8 (12.7)	19 (15.5)	15 (13.6)	8 (11.0)	28 (17.3)	32 (15.6)	5 (12.8)
*P* value	0.53		0.69		0.24		0.39	
***CREB1***								
GG	48 (33.1)	13 (20.6)	36 (29.3)	28 (25.4)	23 (31.5)	41 (25.3)	57 (27.8)	11 (28.2)
GA or AA	97 (66.9)	50 (79.4)	87 (70.7)	82 (74.6)	50 (68.5)	121 (74.7)	148 (72.2)	28 (71.8)
*P* value	0.07		0.51		0.32		0.95	
GG or GA	122 (84.1)	43 (68.3)	100 (81.3)	79 (71.8)	63 (86.3)	117 (72.2)	156 (76.1)	32 (82.0)
AA	23 (15.9)	20 (31.7)	23 (18.7)	31 (28.2)	10 (13.7)	45 (27.8)	49 (23.9)	7 (18.0)
*P* value	0.009**		0.08		0.012**		0.53	
***MITF***								
GG	43 (29.6)	14 (32.6)	36 (29.3)	30 (22.7)	25 (34.7)	41 (25.3)	62 (30.2)	6 (15.4)
GA or AA	102 (70.4)	49 (67.4)	87 (70.7)	80 (77.3)	48 (65.3)	121 (74.7)	143 (69.8)	33 (84.6)
*P* value	0.26		0.15		0.73		0.07	
GG or GA	106 (73.1)	42 (66.7)	93 (75.6)	76 (69.1)	52 (71.2)	119 (73.5)	154 (75.1)	21 (53.9)
AA	39 (26.9)	21 (33.3)	30 (24.4)	34 (30.9)	21 (28.8)	43 (26.5)	51 (24.9)	18 (46.1)
*P* value	0.34		0.72		0.26		0.007**	

### *ADCY3*, *CREB1* and *MITF* expression

Similar mRNA expressions (in arbitrary units ± standard deviation) were seen in CM patients with distinct genotypes of *ADCY3* (TT: 1.13 ± 0.55, TG: 0.90 ± 0.66, GG: 1.16 ± 0.85; *p* = 0.52) (Figure S1A Supplement), *CREB1* (GG: 1.21 ± 0.76, GA: 1.19 ± 0.79, AA: 1.14 ± 0.63; *p* = 0.98) (Figure S1B Supplement), and *MITF* (GG: 1.08 ± 0.47, GA: 0.98 ± 0.74, AA: 1.03 ± 0.61; *p* = 0.91) (Figure S1C Supplement). Expressions of mRNA were also similar in controls with distinct genotypes of *ADCY3* (TT: 1.06 ± 0.34, TG: 1.06 ± 0.44, GG: 1.08 ± 0.74; *p* = 0.98) (Figure S1D Supplement), *CREB1* (GG: 1.06 ± 0.42, GA: 1.24 ± 0.78, AA: 1.48 ± 0.70; *p* = 0.29) (Figure S1E Supplement), and *MITF* (GG: 1.15 ± 0.69, GA: 1.24 ± 0.45, AA: 0.99 ± 0.46; *p* = 0.29) (Figure S1F Supplement).

### Association of clinicopathological aspects and genotypes with patients’ survival

We obtained consisted survival data from 210 CM patients. The median follow-up time of patients enrolled in the survival analysis was 97 months (range 5–228 months). The patient’s final status was established on January 2020, when 136 patients were alive (132 without disease, 4 with disease) and 74 patients had died (46 due to disease, 28 of unrelated causes).

At 60 months of follow-up, the PFS was lower in males (68.5% versus 81.3%, *p* = 0.02), patients with tumors located in head or trunk (70.9% versus 82.0%, *p* = 0.03), patients with tumor with Breslow index higher 1.5 mm (54.9% versus 94.4%, *p* < 0.0001), Clark levels III to V (66.3% versus 94.1%, *p* < 0.0001) and III or IV stage (32.3% versus 82.2%, *p* < 0.0001) (Kaplan–Meier estimates). Differences among groups remained the same in univariate analysis. In multivariate analysis, CM located in head or trunk (HR: 2.38), thicker tumors (HR: 4.93), stage III or IV tumors (HR: 3.30), and *ADCY3* TT genotype (HR: 1.89) (Fig. 1B) were predictors of poor PFS. At 60 months of follow-up, the MSS was lower in males (76.4% versus 93.7%, *p* < 0.0001), patients with tumors with Breslow index higher 1.5 mm (72.8% versus 97.1%, *p* < 0.0001), Clark levels III to V (79.1% versus 98.5%, *p* < 0.0001) and stage III or IV (45.2% versus 91.3%, *p* < 0.0001), and *MITF* GA or AA genotype (81.9% versus 91.3%, *p* = 0.04) (Fig. 1C) (Kaplan–Meier estimates). Differences among groups remained the same in univariate analysis; patients with *MITF* GA or AA genotype had 2.20 more chances of evolving to death by CM than others. In multivariate analysis, males (HR: 3.12), thicker tumors (HR: 4.86) and III or IV tumor stage (HR: 4.01) were predictors of poor MMS (Table 4).

**Table 4:** Clinicopathological aspects and genotypes in survival of 210 cutaneous melanoma patients. HR, hazard ratio; CI, confidence interval; NC, characteristic not computed in multivariate analysis. ^*^The total numbers of individuals differed from the total quoted because it was not possible to obtain consistent information about characteristics in some individuals. ^a^*P*_bootstrap_ = 0.01; ^b^*P*_bootstrap_ = 0.001. ^c^*P*_bootstrap_ = 0.001. ^d^*P*_bootstrap_ = 0.02; ^e^*P*_bootstrap_ = 0.002. ^f^*P*_bootstrap_ = 0.006. ^g^*P*_bootstrap_ < 0.0001 in multivariate analysis. Significant differences between groups are presented in bold letters.

Variable	Progression-free survival					Melanoma-specific survival				
	N total/N events	Univariate Cox analysis		Multivariate Cox analysis		N total/ N events	Univariate Cox analysis		Multivariate Cox analysis	
		HR (95% CI)	*p*	HR (95% CI)	*p*		HR (95% CI)	*P*	HR (95% CI)	*p*
**Median age**										
≤ 55 years	102/31	Reference	0.50	Reference	0.17	102/21	Reference	0.50	NA	NA
> 55 years	108/36	1.18 (0.72–1.91)		1.40 (0.86–2.29)		108/25	1.22 (0.68–2.18)			
**Gender**										
Male	113/43	1.78 (1.07–2.94)	**0.02**	1.09 (0.63–1.88)	0.74	113/36	3.61 (1.79–7.29)	** < 0.001**	3.12 (1.52–6.41)	**0.002**^**e**^
Female	97/24	Reference		Reference		97/10	Reference		Reference	
**Tumor location**										
Head or trunk	143/52	1.83 (1.03–3.26)	**0.03**	2.38 (1.22–4.62)	**0.01**^**a**^	143/35	1.54 (0.78–3.03)	0.21	NA	NA
Upper/lower limb	67/15	Reference		Reference		67/11	Reference			
Breslow thickness*										
≤ 1.5 mm	109/12	Reference	** < 0.001**	Reference	** < 0.001**^**b**^	109/7	Reference	** < 0.001**	Reference	** < 0.001**^**f**^
> 1.5 mm	90/47	6.51 (3.44–12.29)		4.93 (2.53–9.57)		90/33	6.64 (2.93–15.04)		4.86 (2.09–11.31)	
Clark level*										
I or II	69/6	Reference	** < 0.001**	Reference	0.20	69/3	Reference	**0.001**	Reference	0.29
III-V	132/55	5.71 (2.45–13.29)		1.89 (0.70–5.05)		132/38	7.39 (2.28–23.96)		2.08 (0.53–8.12)	
TNM stage*										
0-II	177/43	Reference	** < 0.001**	Reference	** < 0.001**^**c**^	177/26	Reference	** < 0.001**	Reference	** < 0.001**^** g**^
III or IV	31/23	5.18 (3.08–8.71)		3.30 (1.85–5.89)		31/20	7.51 (4.17–13.56)		4.01 (2.07–7.74)	
***ADCY3***										
TT	74/29	1.41 (0.87–2.29)	0.16	1.89 (1.11–3.21)	**0.01**^**d**^	74/21	1.58 (0.88–2.83)	0.12	1.49 (0.79–2.82)	0.21
TG or GG	136/38	Reference		Reference		136/25	Reference		Reference	
TT or TG	180/58	1.04 (0.51–2.12)	0.89	NC	NC	180/40	1.06 (0.42–2.37)	0.99	NC	NA
GG	30/9	Reference				30/6	Reference			
***CREB1***										
GG	60/15	Reference	0.19	Reference	0.23	60/10	Reference	0.32	NC	NA
GA or AA	150/52	1.51 (0.87–2.63)		1.45 (0.78–2.70)		150/36	1.42 (0.70–2.87)			
GG or GA	164/49	Reference	0.19	Reference	0.28	164/34	Reference	0.50	NC	NA
AA	46/18	1.43 (0.83–2.46)		1.37 (0.76–2.47)		46/12	1.24 (0.64–2.41)			
***MITF***										
GG	59/14	Reference	0.22	NC	NC	59/7	Reference	**0.05**	Reference	0.19
GA or AA	151/53	1.44 (0.80–2.60)				151/39	2.20 (1.00–4.93)		1.79 (0.74–4.34)	
GG or GA	150/45	Reference	0.25	NC	NC	150/30	Reference	0.22	NC	NC
AA	60/22	1.34 (0.80–2.23)				60/16	1.45 (0.79–2.66)			

## Discussion

In this study, we investigated and identified intronic SNVs *ADCY3* c.675+9196T>G, *CREB1* c.303+373G>A, and *MITF* c.938-325G>A in pigmentation-related genes in association with CM risk and clinicopathological features.

After screening SNVs (stage 1), we found more than 6,000 SNVs associated with CM risk in introns of genes, according to previous studies^14–18^, and we selected three SNVs involved in the splicing regulatory sequences of pigmentation-related genes for data validation, due to their potential roles in determining abnormalities in production and/or function of the respective encoded proteins^13,14^.

In fact, previous GWAS have shown that the majority of disease-associated variants reside in the non-coding regions of the genome, suggesting that gene regulatory changes contribute to disease risk^18^. On the other hand, splicing comprises a two-step reaction of intron removal and exon ligation and is essential for gene expression: pre-mRNA splicing is catalyzed by the spliceosome, a large complex of ribonucleoproteins (RNPs), and this complex recognizes the target sequences and assembles on pre-mRNA^20^.

After SNVs validation (stage 2), we observed that *CREB1* GA or AA genotype and allele “A” were more common in CM patients than in controls, and that individuals with referred genotypes and allele were under 1.79 and 1.47-fold increased risks of CM than others, respectively.

*CREB1* was highly expressed in tumor cells, such as human gastric cell lines and knockdown of *CREB1* inhibited human gastric cancer cells growth^38^. *CREB1* has also been seen as an important gene in CM development^8^, and analysis of common network from cancer type-specific RNA-Seq co-expression data showed *CREB1* as a melanoma-associated gene^39^. To the best of our knowledge, there are no previous studies focusing the roles of *ADCY3* c.675+9196T>G, *CREB1* c.303+373G>A, and *MITF* c.938-325G>A SNVs in risk of CM, and therefore the association of *CREB1* GA or AA genotype and allele “A” with CM risk seen in the present study is a new finding. The search for potential splicing regulatory elements using in silico algorithm in this study indicated that gene variants induce the creation or abrogation of binding sites^33^. The allele “A” of *CREB1* c.303+373G>A may alter binding sites for splicing elements, such as the hnRNP A1, and possibly increases *CREB1* activity due to altered efficiency of splicing^19,20,33^. Since *CREB1* is a transcription factor that stimulates the *MITF* activity, the increase of its activity may in turn increase *MITF* activity, having proliferation of abnormal melanocytes and increased risk for CM as consequence^8^.

When genotypes were analyzed in patients stratified by clinicopathological aspects, we noted that *CREB1* AA variant genotype was more common in patients with tumors located in limbs than in patients with tumors located in head or trunk and with tumors at Clark level III to IV than in patients with tumors at I or II level. In addition, an excess of *MITF* AA genotype was found in patients with tumors at stage III or IV than in those with stage I or II tumors.

It was already described that acquisition of metastatic phenotype in CM involved the gain in expression of CREB/activating transcription factor-1 (CREB/ATF-1)^40^ and *MITF* amplification^41^. However, how far our knowledge reaches, this study is the first to describe the influence of *CREB1* c.303+373G>A and *MITF* c.938-325G>A SNVs on clinicopathological features of CM. Indeed, *CREB1* promotes tumorigenesis by increasing cell migration, proliferation, and invasiveness, through its effects on the MITF pathway^8^. The in silico analysis showed that the variant allele “A” of *MITF* c.938-325G>A may create a site of ligation for splicing factors, including the hnRNP A1, possibly determining increase in gene expression^33^. Thus, we postulate that *CREB1* AA and *MITF* AA genotypes may increase abnormal melanocytes proliferation and consequently improve aggressiveness of CM.

We also noted that *ADCY3* c.675+9196 TT genotype was associated with shorter PFS while *MITF* GA or AA genotype was associated with shorter MSS in CM patients, when compared to the remaining genotypes.

Up-regulation of *ADCY3* increased the tumorigenic potential of gastric cells^42^ and predicted shorter overall survival in patients with pancreatic cancer^43^. Overexpression of ADCY2 was previously associated with aggressive behavior of CM^44^, and MITF amplification predicted worst survival of CM patients^41^. The in silico analysis showed that the *ADCY3* c.675+9196T>G variant may alter sites of ligation for splicing factors, including the SRp55 and 9G8, with a possible increase in the efficiency of splicing and gene expression^19,20,33^. Since ADCYs participate in *CREB* activation, and *CREB* regulates the expression of *MITF*^8^, the increase in *ADCY3* activity in CM patients with the TT genotype may favor proliferation of abnormal melanocytes leading to relapse or death by CM effects. Again, the possible increased activity of *MITF* in patients with GA or AA genotype may have contributed to this clinical unfavorable outcome.

It is also worth to comment that pigmentation-related genes have been seen as potential therapeutic targets. Previous studies showed that increased *ADCY* expression generated resistance to MAPK inhibitions and up regulates *MITF* in melanoma cells^8^, and the suppression of *MITF* expression by the CH6868398 agent caused melanoma cell growth inhibition^45^. Inhibition of p300 acetyltransferase transcriptional coactivator of MITF by p300/CBP complex had growth inhibitory effects in melanoma cells expressing MITF^46,47^, and Kazinol U reduced melanogenesis by inhibition of MITF in melanoma cells^48^. Since response to new agents depends on *ADCY3* and *MITF* expressions, it is possible that patients with distinct genotypes of these genes present differentiated responses to therapies.

At this time, we draw attention to the fact that no differences in *ADCY3*, *CREB1* and *MITF* expressions were identified in leukocytes of peripheral blood of individuals with the distinct genotypes of *ADCY3*, *CREB1* and *MITF* SNVs. It is possible that the sample size evaluated was not enough to identify differences in gene expression among individuals or, alternatively, these variants may determine gene expression abnormalities only in tumor tissue or only protein functional changes.

In summary, we described for the first time the potential importance of *ADCY3* c.675+9196T>G, *CREB1* c.303+373G>A, and *MITF* c.938-325G>A SNVs in the pigmentation-related genes in CM risk and clinicopathological features in Brazilian individuals. We recognize that the present study has limitations: it was conducted on a relatively small number of individuals and only quantitative analysis of gene expression in normal leukocytes was performed. Thus, we believe that our results will require confirmation in a further larger epidemiological study in our population and others, and quantitative and functional analyses of *ADCY3*, *CREB1* and *MITF* SNVs in melanoma cells. If these findings are confirmed, they might help to identify individuals with high risk for CM who deserves to receive additional recommendations for CM prevention and early tumor detection and/or differentiated treatment, perhaps including the targeting lineage specific MC1R signalizing pathway agents.

## Supplementary information

Supplementary Information

## Data Availability

The authors declare that all data of the present study are available for the corresponding author upon reasonable request.
