# Supplementary material for: Inherited variations in human pigmentation-related genes modulate cutaneous melanoma risk and clinicopathological features in Brazilian population
Source: Sci Rep. 2020 Jul 22;10:12129. doi: 10.1038/s41598-020-68945-9 (PMC7376158; doi:10.1038/s41598-020-68945-9)
Supplement: Supplementary file 1 — Supplementary Information [file 41598_2020_68945_MOESM1_ESM.doc]

**Supplement**

**Inherited variations in human pigmentation-related genes modulate cutaneous melanoma risk and clinicopathological features in Brazilian population**

Gustavo Jacob Lourenço^1,†^, Cristiane Oliveira^1,†^, Benilton Sá Carvalho^2^, Caroline Torricelli^1^, Janet Keller Silva^1^, Gabriela Vilas Bôas Gomez^1^, José Augusto Rinck-Junior^3,4^, Wesley Lima Oliveira^1^, Vinicius Lima Vazquez^5^, Sergio Vicente Serrano^6^, Aparecida Machado Moraes^3^, Carmen Silvia Passos Lima^1,3^

^1^Laboratory of Cancer Genetics, Faculty of Medical Sciences, University of Campinas, Campinas, São Paulo, Brazil

^2^Department of Statistics, Institute of Mathematics, Statistic, and Computer Science, University of Campinas, Campinas, São Paulo, Brazil

^3^Department of Internal Medicine, Faculty of Medical Sciences, University of Campinas, Campinas, São Paulo, Brazil

^4^A.C. Camargo Cancer Center, São Paulo, São Paulo, Brazil

^5^Melanoma and Sarcoma Surgery Department, Barretos Cancer Hospital, Barretos, São Paulo, Brazil

^6^Department of Medical Oncology, Barretos Cancer Hospital, Barretos, São Paulo, Brazil

^†^These authors contributed equally to this work

**Table S1**. Single nucleotide variants identified in 103 patients with cutaneous melanoma and 103 controls using Affymetrix Genome-Wide Human SNV Arrays 6.0.

| **Probe number** | **SNV rs** | **Gene symbol** | **OR** | **95% CI** | | ***p* value** |
| --- | --- | --- | --- | --- | --- | --- |
| SNP_A-2172006 | rs2586777 | *MRO* | 0.15 | 0.07 | 0.33 | 0.00000216 |
| SNP_A-2252151 | rs512743 | Intergenic | 0.17 | 0.08 | 0.37 | 0.00000804 |
| SNP_A-1946972 | rs4677905 | *MYLK* | 5.76 | 2.67 | 12.45 | 0.00000831 |
| SNP_A-4236286 | rs2849233 | *MRO* | 0.17 | 0.08 | 0.37 | 0.00000849 |
| SNP_A-2043259 | rs2072885 | *TENM1* | 0.15 | 0.07 | 0.35 | 0.00000944 |
| SNP_A-4201200 | rs284732 | Intergenic | 0.19 | 0.09 | 0.40 | 0.00001164 |
| SNP_A-8481275 | rs4823473 | *TBC1D22A* | 7.03 | 2.93 | 16.83 | 0.00001211 |
| SNP_A-8675796 | rs13183333 | Intergenic | 5.68 | 2.59 | 12.45 | 0.00001409 |
| SNP_A-2281073 | rs9827237 | *MYLK* | 5.23 | 2.47 | 11.06 | 0.00001516 |
| SNP_A-1803305 | rs284723 | Intergenic | 0.20 | 0.09 | 0.41 | 0.00001612 |
| SNP_A-1809072 | rs5766701 | *TBC1D22A* | 6.83 | 2.84 | 16.41 | 0.00001751 |
| SNP_A-8541945 | rs10043564 | *ADGRV1* | 5.33 | 2.47 | 11.47 | 0.00001925 |
| SNP_A-8649249 | rs4928 | *MCM7* | 0.15 | 0.06 | 0.35 | 0.00001976 |
| SNP_A-1995171 | rs1430324 | Intergenic | 6.16 | 2.67 | 14.22 | 0.00002003 |
| SNP_A-8348351 | rs10956600 | Intergenic | 0.16 | 0.07 | 0.37 | 0.00002003 |
| SNP_A-4226938 | rs9918761 | Intergenic | 0.16 | 0.07 | 0.37 | 0.00002003 |
| SNP_A-8331228 | rs12733192 | Intergenic | 4.88 | 2.34 | 10.18 | 0.00002359 |
| SNP_A-8445817 | rs12449029 | *RBFOX1* | 0.21 | 0.10 | 0.44 | 0.00002410 |
| SNP_A-4225759 | rs512660 | Intergenic | 0.20 | 0.09 | 0.42 | 0.00002450 |
| SNP_A-2058843 | rs2212852 | Intergenic | 5.06 | 2.37 | 10.79 | 0.00002673 |
| SNP_A-4204631 | rs1074258 | Intergenic | 0.20 | 0.09 | 0.42 | 0.00002760 |
| SNP_A-2057422 | rs12596244 | *ADAMTS18* | 7.99 | 3.01 | 21.18 | 0.00002971 |
| SNP_A-4280973 | rs7299057 | *TMEM132D* | 0.14 | 0.06 | 0.35 | 0.00003067 |
| SNP_A-8518924 | rs517633 | Intergenic | 0.20 | 0.09 | 0.43 | 0.00003201 |
| SNP_A-2036037 | rs1526377 | Intergenic | 0.20 | 0.09 | 0.43 | 0.00003274 |
| SNP_A-8690334 | rs10857975 | Intergenic | 0.21 | 0.10 | 0.44 | 0.00003280 |
| SNP_A-8664988 | rs400065 | *ADAMTS19* | 0.21 | 0.10 | 0.44 | 0.00003381 |
| SNP_A-2063564 | rs10916711 | *PLA2G2D* | 0.21 | 0.10 | 0.44 | 0.00003482 |
| SNP_A-2075758 | rs7279678 | Intergenic | 5.75 | 2.50 | 13.20 | 0.00003778 |
| SNP_A-8434066 | rs10445082 | *ADAMTS18* | 8.28 | 3.03 | 22.64 | 0.00003821 |
| SNP_A-1828631 | rs881301 | Intergenic | 0.22 | 0.10 | 0.45 | 0.00003822 |
| SNP_A-2308582 | rs10087659 | Intergenic | 0.16 | 0.07 | 0.39 | 0.00004224 |
| SNP_A-8455018 | rs12598504 | *ADAMTS18* | 8.22 | 3.00 | 22.52 | 0.00004268 |
| SNP_A-8611908 | rs10845690 | *GPRC5D-AS1* | 4.51 | 2.18 | 9.33 | 0.00004979 |
| SNP_A-2272666 | rs4823474 | *TBC1D22A* | 5.33 | 2.37 | 11.99 | 0.00005318 |
| SNP_A-8381423 | rs1428739 | Intergenic | 4.71 | 2.22 | 10.01 | 0.00005480 |
| SNP_A-8341425 | rs2966368 | *PRRC2B* | 0.12 | 0.04 | 0.34 | 0.00005543 |
| SNP_A-1812396 | rs17637167 | Intergenic | 0.23 | 0.11 | 0.47 | 0.00005595 |
| SNP_A-8456901 | rs6720545 | Intergenic | 0.18 | 0.08 | 0.42 | 0.00005618 |
| SNP_A-8373339 | rs2919045 | *AP003066.1* | 4.61 | 2.19 | 9.71 | 0.00005672 |
| SNP_A-8583088 | rs7724804 | Intergenic | 0.23 | 0.11 | 0.47 | 0.00005733 |
| SNP_A-8540673 | rs2270953 | *DCC* | 0.19 | 0.09 | 0.43 | 0.00005805 |
| SNP_A-2121707 | rs2455144 | *PRDM16* | 4.95 | 2.26 | 10.80 | 0.00006073 |
| SNP_A-4257654 | rs1074983 | Intergenic | 0.22 | 0.11 | 0.46 | 0.00006235 |
| SNP_A-2297944 | rs4375766 | Intergenic | 4.54 | 2.16 | 9.51 | 0.00006235 |
| SNP_A-8477373 | rs2576253 | *ESRRG* | 4.68 | 2.20 | 9.99 | 0.00006366 |
| SNP_A-8280053 | rs17200703 | Intergenic | 0.18 | 0.08 | 0.42 | 0.00006403 |
| SNP_A-1890945 | rs7175624 | *AC021351.1* | 4.74 | 2.21 | 10.19 | 0.00006727 |
| SNP_A-8481096 | rs13026352 | Intergenic | 4.88 | 2.24 | 10.63 | 0.00006793 |
| SNP_A-1943502 | rs41505152 | *AC058822.1* | 0.18 | 0.08 | 0.42 | 0.00006940 |
| SNP_A-8487904 | rs17416013 | Intergenic | 5.18 | 2.30 | 11.64 | 0.00006984 |
| SNP_A-2031860 | rs10828545 | *CACNB2* | 0.23 | 0.11 | 0.47 | 0.00007079 |
| SNP_A-8666752 | rs3861395 | *AL592429.2* | 0.21 | 0.10 | 0.46 | 0.00007373 |
| SNP_A-1837788 | rs509566 | *GRIK4* | 5.63 | 2.39 | 13.23 | 0.00007399 |
| SNP_A-4222659 | rs1349711 | *FAM172A* | 4.52 | 2.14 | 9.54 | 0.00007511 |
| SNP_A-8381262 | rs1239826 | Intergenic | 6.93 | 2.66 | 18.08 | 0.00007585 |
| SNP_A-2103218 | rs4835975 | *ADAMTS19* | 0.22 | 0.10 | 0.46 | 0.00007679 |
| SNP_A-2220559 | rs4941815 | Intergenic | 11.83 | 3.48 | 40.26 | 0.00007715 |
| SNP_A-8433685 | rs7933978 | *NAV2* | 4.71 | 2.18 | 10.17 | 0.00007957 |
| SNP_A-8503574 | rs2756374 | *RNGTT* | 0.14 | 0.05 | 0.37 | 0.00008034 |
| SNP_A-1818024 | rs4890914 | *AC100863.1* | 0.19 | 0.09 | 0.44 | 0.00008039 |
| SNP_A-2145157 | rs1494775 | Intergenic | 4.24 | 2.06 | 8.71 | 0.00008480 |
| SNP_A-2140037 | rs4891573 | Intergenic | 4.11 | 2.03 | 8.33 | 0.00008537 |
| SNP_A-2057122 | rs7114011 | *NUP160* | 4.12 | 2.03 | 8.35 | 0.00008612 |
| SNP_A-1981239 | rs26200 | Intergenic | 5.18 | 2.28 | 11.79 | 0.00008624 |
| SNP_A-8664879 | rs26203 | Intergenic | 5.18 | 2.28 | 11.79 | 0.00008624 |
| SNP_A-8454358 | rs1880897 | Intergenic | 7.30 | 2.70 | 19.71 | 0.00008757 |
| SNP_A-2156000 | rs5915451 | Intergenic | 0.14 | 0.05 | 0.37 | 0.00008757 |
| SNP_A-8531316 | rs7659353 | Intergenic | 5.60 | 2.36 | 13.30 | 0.00009360 |
| SNP_A-8356013 | rs7520918 | *KCNN3* | 0.21 | 0.09 | 0.46 | 0.00009413 |
| SNP_A-8516116 | rs10085679 | *ZNF804B* | 4.60 | 2.14 | 9.91 | 0.00009423 |
| SNP_A-8346968 | rs9418803 | *DOCK1* | 4.27 | 2.06 | 8.86 | 0.00009607 |
| SNP_A-2117901 | rs4665628 | *KLHL29* | 0.21 | 0.09 | 0.46 | 0.00009633 |
| SNP_A-4230297 | rs9582776 | Intergenic | 4.38 | 2.08 | 9.21 | 0.00009771 |
| SNP_A-2292834 | rs9420502 | *RPS7P9* | 0.16 | 0.06 | 0.40 | 0.00009782 |
| SNP_A-1876945 | rs9586343 | Intergenic | 0.23 | 0.11 | 0.48 | 0.00009897 |

SNV: single nucleotide variant. rs: reference number. OR: odds ratio. CI: confidence interval.

**Table S2**. Seventy-four single nucleotide variants in 28 human pigmentation-related genes identified by Database for Annotation, Visualization, and Integrated Discovery (david.ncifcrf.gov) and Kyoto Encyclopedia of Genes and Genomes pathway maps (www.kegg.jp).

| **SNV rs** | **Gene symbol** |  | **SNV rs** | **Gene symbol** |  | **SNV rs** | **Gene symbol** |
| --- | --- | --- | --- | --- | --- | --- | --- |
| rs1864071 | *ADCY2* |  | rs982950 | *CREB5* |  | rs1806448 | *PRKCA* |
| rs7567997 | *ADCY3* |  | rs34143286 | *CREB5* |  | rs1806448 | *PRKCA* |
| rs2384058 | *ADCY3* |  | rs4722839 | *CREB5* |  | rs12445719 | *PRKCB* |
| rs2033653 | *ADCY3* |  | rs2237364 | *CREB5* |  | rs6760363 | *PRKCE* |
| rs1865689 | *ADCY3* |  | rs10503830 | *FZD3* |  | rs1868388 | *PRKCE* |
| rs10200566 | *ADCY3* |  | rs2079162 | *GNAI1* |  | rs1536014 | *PRKCH* |
| rs10198275 | *ADCY3* |  | rs17724988 | *GNAQ* |  | rs11852192 | *PRKCH* |
| rs6545814 | *ADCY3* |  | rs6100260 | *GNAS* |  | rs11627926 | *PRKCH* |
| rs11900505 | *ADCY3* |  | rs2017472 | *KIT* |  | rs2058716 | *PRKD3* |
| rs9841477 | *ADCY5* |  | rs10865653 | *MITF* |  | rs4935222 | *PRKG1* |
| rs7812946 | *ADCY8* |  | rs9822057 | *MITF* |  | rs293332 | *PRKG1* |
| rs6997554 | *ADCY8* |  | rs7623610 | *MITF* |  | rs10997502 | *PRKG1* |
| rs10779935 | *CALM2* |  | rs2064272 | *PLCB1* |  | rs1033969 | *PRKG1* |
| rs1027478 | *CALM2* |  | rs17365739 | *PLCB1* |  | rs10997540 | *PRKG1* |
| rs4724298 | *CAMK2B* |  | rs4295085 | *PLCB1* |  | rs10997689 | *PRKG1* |
| rs6853484 | *CAMK2D* |  | rs7272444 | *PLCB4* |  | rs7099012 | *PRKG1* |
| rs28599641 | *CAMK2D* |  | rs2299680 | *PLCB4* |  | rs16923827 | *PRKG1* |
| rs10932201 | *CREB1* |  | rs2727554 | *PRKAG2* |  | rs10953363 | *PRKRIP1* |
| rs11977277 | *CREB3L2* |  | rs2727553 | *PRKAG2* |  | rs2410988 | *PRKRIP1* |
| rs273988 | *CREB3L2* |  | rs10277859 | *PRKAG2* |  | rs7797435 | *PRKRIP1* |
| rs273995 | *CREB3L2* |  | rs2024266 | *PRKAG2* |  | rs2568210 | *TCF7L1* |
| rs273996 | *CREB3L2* |  | rs9632641 | *PRKAG2* |  | rs3790608 | *WNT2B* |
| rs10279584 | *CREB5* |  | rs11772236 | *PRKAG2* |  | rs1417823 | *WNT8B* |
| rs17156685 | *CREB5* |  | rs11766003 | *PRKAG2* |  | rs10883497 | *WNT8B* |
| rs41342 | *CREB5* |  | rs12601850 | *PRKCA* |  |  |  |

SNV: single nucleotide variant. rs: reference number.

**Table S3**. Seventy-four single nucleotide variants located in 28 human pigmentation-related genes identified by Database for Annotation, Visualization, and Integrated Discovery (david.ncifcrf.gov) and Kyoto Encyclopedia of Genes and Genomes pathway maps (www.kegg.jp) and *in silico* analysis for identification of splicing regulatory sequences.

| **SNV rs** | **Gene symbol** | **Splicing motifs algorithms^*^** | | | |
| --- | --- | --- | --- | --- | --- |
|  |  | **Splicing site** | **Branch point site** | **Enhancer motifs** | **Silencer motifs** |
| rs1864071 | *ADCY2* | ND | ND | ND | Broken |
| rs7567997 | *ADCY3* | ND | ND | ND | ND |
| rs2384058 | *ADCY3* | New acceptor | New | ND | Broken |
| rs2033653 | *ADCY3* | New donor | ND | New (SF2) | ND |
| rs1865689 | *ADCY3* | New donor | ND | Broken (SRp40) | Broken |
| rs10200566 | *ADCY3* | ND | ND | ND | ND |
| rs10198275 | *ADCY3* | New donor | ND | New (SRp40) | ND |
| rs6545814 | *ADCY3* | ND | ND | ND | New |
| **rs11900505** | ***ADCY3*** | **New acceptor** | **Broken** | **New (SRp55)** | **New** |
| rs9841477 | *ADCY5* | ND | ND | ND | ND |
| rs7812946 | *ADCY8* | ND | Broken | New (SC35) | Broken |
| rs6997554 | *ADCY8* | New acceptor | ND | New (SF2) | Broken |
| rs10779935 | *CALM2* | ND | ND | ND | ND |
| rs1027478 | *CALM2* | ND | ND | ND | ND |
| rs4724298 | *CAMK2B* | ND | ND | New (9G8) | Broken |
| rs6853484 | *CAMK2D* | ND | ND | New (SC35) | Broken |
| rs28599641 | *CAMK2D* | ND | ND | New (SRp40) | Broken |
| **rs10932201** | ***CREB1*** | **Broken donor** | **New** | **New (SRp55)** | **Broken** |
| rs11977277 | *CREB3L2* | ND | ND | ND | ND |
| rs273988 | *CREB3L2* | New donor | ND | Broken (SRp55) | ND |
| rs273995 | *CREB3L2* | ND | ND | ND | Broken |
| rs273996 | *CREB3L2* | ND | ND | ND | ND |
| rs10279584 | *CREB5* | ND | ND | ND | ND |
| rs17156685 | *CREB5* | ND | ND | ND | ND |
| rs41342 | *CREB5* | ND | ND | ND | ND |
| rs982950 | *CREB5* | ND | ND | ND | ND |
| rs34143286 | *CREB5* | ND | ND | ND | ND |
| rs4722839 | *CREB5* | ND | ND | ND | ND |
| rs2237364 | *CREB5* | ND | ND | ND | ND |
| rs10503830 | *FZD3* | New donor | ND | ND | Broken |
| rs2079162 | *GNAI1* | ND | ND | ND | ND |
| rs17724988 | *GNAQ* | ND | ND | ND | ND |
| rs6100260 | *GNAS* | ND | ND | ND | ND |
| rs2017472 | *KIT* | ND | ND | ND | ND |
| rs10865653 | *MITF* | ND | ND | ND | ND |
| rs9822057 | *MITF* | ND | ND | ND | ND |
| **rs7623610** | ***MITF*** | **New donor** | **Broken** | **Broken** | **New** |
| rs2064272 | *PLCB1* | ND | ND | ND | ND |
| rs17365739 | *PLCB1* | ND | ND | New | New |
| rs4295085 | *PLCB1* | ND | ND | ND | ND |
| rs7272444 | *PLCB4* | New donor | ND | New (SRp55) | ND |
| rs2299680 | *PLCB4* | ND | ND | ND | ND |
| rs2727554 | *PRKAG2* | New acceptor | ND | ND | ND |
| rs2727553 | *PRKAG2* | ND | ND | New (9G8) | Broken |
| rs10277859 | *PRKAG2* | ND | ND | New (9G8) | Broken |
| rs2024266 | *PRKAG2* | ND | ND | New | ND |
| rs9632641 | *PRKAG2* | New donor | ND | New | ND |
| rs11772236 | *PRKAG2* | ND | ND | New (SC35) | Broken |
| rs11766003 | *PRKAG2* | ND | ND | ND | ND |
| rs12601850 | *PRKCA* | ND | ND | ND | ND |
| rs1806448 | *PRKCA* | ND | ND | ND | ND |
| rs1806448 | *PRKCA* | ND | ND | ND | ND |
| rs12445719 | *PRKCB* | ND | ND | New (SRp40) | ND |
| rs6760363 | *PRKCE* | ND | ND | New (9G8) | Broken |
| rs1868388 | *PRKCE* | New donor | ND | ND | ND |
| rs1536014 | *PRKCH* | ND | ND | ND | ND |
| rs11852192 | *PRKCH* | New acceptor | ND | ND | ND |
| rs11627926 | *PRKCH* | ND | ND | ND | ND |
| rs2058716 | *PRKD3* | ND | ND | ND | ND |
| rs4935222 | *PRKG1* | ND | ND | ND | Broken |
| rs293332 | *PRKG1* | ND | ND | ND | ND |
| rs10997502 | *PRKG1* | ND | ND | New | Broken |
| rs1033969 | *PRKG1* | ND | ND | New | Broken |
| rs10997540 | *PRKG1* | ND | ND | New | Broken |
| rs10997689 | *PRKG1* | ND | ND | ND | ND |
| rs7099012 | *PRKG1* | New donor | ND | New (SRp40) | Broken |
| rs16923827 | *PRKG1* | ND | ND | ND | ND |
| rs10953363 | *PRKRIP1* | ND | ND | New (SF2) | ND |
| rs2410988 | *PRKRIP1* | ND | ND | New (SRp40) | ND |
| rs7797435 | *PRKRIP1* | ND | ND | ND | ND |
| rs2568210 | *TCF7L1* | ND | ND | ND | ND |
| rs3790608 | *WNT2B* | ND | ND | ND | ND |
| rs1417823 | *WNT8B* | ND | ND | ND | ND |
| rs10883497 | *WNT8B* | ND | ND | ND | ND |

SNV: single nucleotide variant. rs: reference number. ^*^Ancestral allele was taken as reference. The algorithms are included in the analysis of Human Splice Finder software (version 3.1) (http://www.umd.be/HSF3/index.html). ND: no difference between single nucleotide variant and reference sequence. SNVs selected for validation are presented in bold letters.

**Table S4**. *ADCY3* c.675+9196T>G, *CREB1* c.303+373G>A, and *MITF* c.938-325G>A combined genotypes in 247 patients with cutaneous melanoma and 280 controls.

| **Genotype** | **Patients n (%)** | **Controls n (%)** | ***P* value  (*P*^c^ value)** | **OR (95% CI)** |
| --- | --- | --- | --- | --- |
| ***ADCY3*** + ***CREB1*** |  |  |  |  |
| GG + GG | 13 (7.8) | 23 (14.9) | 0.05 (0.13) | Reference |
| TG or TT + GA or AA | 154 (92.2) | 131 (85.1) |  | 2.38 (1.00-5.69) |
| GG or TG + GG or GA | 129 (84.3) | 165 (90.2) | 0.40 (0.48) | Reference |
| TT + AA | 24 (15.7) | 18 (9.8) |  | 1.42 (0.61-3.28) |
| ***ADCY3*** + ***MITF*** |  |  |  |  |
| GG + GG | 12 (7.4) | 23 (13.4) | 0.09 (0.13) | Reference |
| TG or TT + GA or AA | 150 (92.6) | 149 (86.6) |  | 2.24 (0.87-5.72) |
| GG or TG + GG or GA | 115 (84.6) | 156 (88.6) | 0.72 (0.72) | Reference |
| TT + AA | 21 (15.4) | 20 (11.4) |  | 1.16 (0.50-2.64) |
| ***CREB1* + *MITF*** |  |  |  |  |
| GG + GG | 21 (14.0) | 37 (24.3) | **0.03** (0.13) | Reference |
| GA or AA + GA or AA | 129 (86.0) | 115 (75.7) |  | 2.19 (1.05-4.55) |
| GA or AA + GA or GA | 139 (88.0) | 184 (95.3) | 0.07 (0.13) | Reference |
| AA + AA | 19 (12.0) | 09 (4.7) |  | 2.50 (0.89-6.97) |

OR: odds ratio adjusted by age, skin color, sun exposure, and number of nevi; CI: confidence interval; *P*^c^ values are *p* values corrected for multiple testing by the false discovery rate test. *P* and *p*^c^ values < 0.05 are presented in bold letters.

**Table S5**. *ADCY3* c.675+9196T>G, *CREB1* c.303+373G>A, and *MITF* c.938-325A>G genotypes in 247 patients with cutaneous melanoma stratified by age, gender, and skin color.

| **Genotypes** | **Age (in years)** | | **Gender** | | **Skin color** | |
| --- | --- | --- | --- | --- | --- | --- |
|  | **≤ 55** | **> 55** | **Male** | **Female** | **White** | **Non-white** |
| ***ADCY3*** |  |  |  |  |  |  |
| TT | 50 (40.0) | 34 (27.9) | 49 (37.7) | 35 (29.9) | 79 (34.2) | 05 (31.2) |
| TG or GG | 75 (60.0) | 88 (72.1) | 81 (62.3) | 82 (70.1) | 152 (65.8) | 11 (68.8) |
| *P* value | 0.06 | | 0.19 | | 1.00 | |
| TT or TG | 110 (88.0) | 99 (81.1) | 115 (88.5) | 94 (80.3) | 196 (84.8) | 13 (81.3) |
| GG | 15 (12.0) | 23 (18.9) | 15 (11.5) | 23 (19.7) | 35 (15.2) | 03 (18.7) |
| *P* value | 0.13 | | 0.07 | | 0.71 | |
| ***CREB1*** |  |  |  |  |  |  |
| GG | 32 (25.6) | 36 (29.5) | 35 (26.9) | 33 (28.2) | 64 (27.7) | 04 (25.0) |
| GA or AA | 93 (74.4) | 86 (70.5) | 95 (73.1) | 84 (71.8) | 167 (72.3) | 12 (75.0) |
| *P* value | 0.49 | | 0.82 | | 1.00 | |
| GG or GA | 93 (74.4) | 96 (78.7) | 101 (77.7) | 88 (75.2) | 175 (75.8) | 14 (87.5) |
| AA | 32 (25.6) | 26 (21.3) | 29 (22.3) | 29 (24.8) | 56 (24.2) | 02 (12.5) |
| *P* value | 0.42 | | 0.64 | | 0.37 | |
| ***MITF*** |  |  |  |  |  |  |
| GG | 42 (33.6) | 29 (23.8) | 38 (29.2) | 33 (28.2) | 64 (27.7) | 07 (43.7) |
| GA or AA | 83 (66.4) | 93 (76.2) | 92 (70.8) | 84 (71.8) | 167 (72.3) | 09 (56.3) |
| *P* value | 0.08 | | 0.85 | | 0.25 | |
| GG or GA | 95 (76.0) | 83 (68.0) | 94 (72.3) | 84 (71.8) | 166 (71.9) | 12 (75.0) |
| AA | 30 (24.0) | 39 (32.0) | 36 (27.7) | 33 (28.2) | 65 (28.1) | 04 (25.0) |
| *P* value | 0.16 | | 0.92 | | 1.00 | |

Values are expressed as number and percentage.

**Table S6**. *ADCY3* c.675+9196T>G, *CREB1* c.303+373G>A, and *MITF* c.938-325A>G genotypes in 247 patients with cutaneous melanoma stratified by phototype, sun exposure, type of sun exposure, and number of nevi.

| **Genotypes** | **Phototype*** | | **Sun exposure*** | | **Type of sun exposure*** | | **Number of nevi*** | |
| --- | --- | --- | --- | --- | --- | --- | --- | --- |
|  | **I or II** | **III to VI** | **Yes** | **No** | **None/intermittent** | **Chronic** | **≤ 50** | **> 50** |
| ***ADCY3*** |  |  |  |  |  |  |  |  |
| TT | 50 (32.5) | 28 (36.4) | 66 (33.7) | 11 (27.5) | 32 (33.3) | 37 (30.6) | 63 (34.4) | 18 (31.0) |
| TG or GG | 104 (67.5) | 49 (63.6) | 130 (66.3) | 29 (72.5) | 64 (66.7) | 84 (69.4) | 120 (65.6) | 40 (69.0) |
| *P* value | 0.55 | | 0.44 | | 0.66 | | 0.63 | |
| TT or TG | 131 (85.1) | 64 (83.1) | 169 (86.2) | 30 (75.0) | 80 (83.3) | 100 (82.6) | 158 (86.3) | 46 (79.3) |
| GG | 23 (14.9) | 13 (16.9) | 27 (13.8) | 10 (25.0) | 16 (16.7) | 21 (17.4) | 25 (13.7) | 12 (20.7) |
| *P* value | 0.70 | | 0.07 | | 0.89 | | 0.19 | |
| ***CREB1*** |  |  |  |  |  |  |  |  |
| GG | 48 (31.2) | 19 (24.7) | 60 (30.6) | 08 (20.0) | 29 (30.2) | 31 (25.6) | 50 (27.3) | 18 (31.0) |
| GA or AA | 106 (68.8) | 58 (75.3) | 136 (69.4) | 32 (80.0) | 67 (69.8) | 90 (74.4) | 133 (72.7) | 40 (69.0) |
| *P* value | 0.30 | | 0.25 | | 0.45 | | 0.58 | |
| GG or GA | 118 (76.6) | 62 (80.5) | 151 (77.0) | 32 (80.0) | 74 (77.1) | 95 (78.5) | 143 (78.1) | 43 (74.1) |
| AA | 36 (23.4) | 15 (19.5) | 45 (23.0) | 08 (20.0) | 22 (22.9) | 26 (21.5) | 40 (21.9) | 15 (25.9) |
| *P* value | 0.50 | | 0.83 | | 0.80 | | 0.52 | |
| ***MITF*** |  |  |  |  |  |  |  |  |
| GG | 44 (28.6) | 22 (28.6) | 54 (27.6) | 13 (32.5) | 30 (31.3) | 35 (28.9) | 50 (27.3) | 19 (32.8) |
| GA or AA | 110 (71.4) | 55 (71.4) | 142 (72.4) | 27 (67.5) | 66 (68.7) | 86 (71.1) | 133 (72.7) | 39 (67.2) |
| *P* value | 1.00 | | 0.52 | | 0.71 | | 0.42 | |
| GG or GA | 106 (68.8) | 60 (77.9) | 143 (73.0) | 26 (65.0) | 72 (75.0) | 85 (70.2) | 128 (69.9) | 44 (75.9) |
| AA | 48 (31.2) | 17 (22.1) | 53 (27.0) | 14 (35.0) | 24 (25.0) | 36 (29.8) | 55 (30.1) | 14 (24.1) |
| *P* value | 0.14 | | 0.30 | | 0.43 | | 0.38 | |

Values are expressed as number and percentage. ^*^The numbers of patients were not the same included in the study (n = 247) because no consistent information could be obtained from some individuals.





**Figure S1**. *ADCY3* c.675+9196T>G, *CREB1* c.303+373G>A, and *MITF* c.938-325A>G single nucleotide variants and gene expressions. Expression levels (bars show group means) were evaluated in blood leukocytes of peripheral blood of cutaneous melanoma patients and controls. Similar mRNA expression levels (in arbitrary units ± standard deviation) were seen in CM patients with distinct genotypes of *ADCY3* (TT: 1.13 ± 0.55, TG: 0.90 ± 0.66, GG: 1.16 ± 0.85; *P* = 0.52) (**A**), *CREB1* (GG: 1.21 ± 0.76, GA: 1.19 ± 0.79, AA: 1.14 ± 0.63; *P* = 0.98) (**B**), and *MITF* (GG: 1.08 ± 0.47, GA: 0.98 ± 0.74, AA: 1.03 ± 0.61; *P* = 0.91) (**C**). Similar mRNA expression levels (in arbitrary units ± standard deviation) were seen in controls with distinct genotypes of *ADCY3* (TT: 1.06 ± 0.34, TG: 1.06 ± 0.44, GG: 1.08 ± 0.74; *P* = 0.98) (**D**), *CREB1* (GG: 1.06 ± 0.42, GA: 1.24 ± 0.78, AA: 1.48 ± 0.70; *P* = 0.29) (**E**), and *MITF* (GG: 1.15 ± 0.69, GA: 1.24 ± 0.45, AA: 0.99 ± 0.46; *P* = 0.29) (**F**).
